# Phenotypic Diversity and Genome-Wide Association Analysis of Decaploid *Camellia hainanica* Germplasm from Hainan Island

**DOI:** 10.3390/ijms27219828

**Published:** 2026-11-02

**Authors:** Taizhen Chen, Jian Yu, Jueqi Chen, Shanzhi Lin, Wuyun Tana, Peng Ling

**Affiliations:** 1 Institute of Plant Protection, Research Center of Quality Safety and Standards for Agro-Products, Hainan Academy of Agricultural Sciences, Haikou 571100, China; ctaizhen@163.com; 2 College of Tropical Agriculture and Forestry, Hainan University, Haikou 570228, China; 3 College of Life Science and Technology, Huazhong Agricultural University, Wuhan 430070, China; jianyu202603@126.com; 4 Spice and Beverage Research Institute, Chinese Academy of Tropical Agricultural Sciences (CATAS), Wanning 571533, China; chenjueqi777@163.com; 5 College of Biological Science and Technology, Beijing Forestry University, Beijing 100083, China; linsz2002@163.com; 6 Research Institute of Non-Timber Forestry, Chinese Academy of Forestry, Zhengzhou 450003, China

**Keywords:** *Camellia hainanica*, genome-wide association study, phenotypic diversity, single-nucleotide polymorphism, whole-genome resequencing

## Abstract

*Camellia hainanica* is an endemic woody oil species from Hainan Island, but the phenotypic and genomic bases of its agronomic and ornamental variation remain poorly understood. Here, 31 phenotypic traits were evaluated in 100 accessions from Changjiang, Hainan, and flower-diameter variation guided the selection of 30 accessions for whole-genome resequencing and genome-wide association analysis. Coefficients of variation ranged from 8.81% to 56.85%, with flower density and first-branch height showing the greatest variation. Cluster analysis separated five groups with contrasting phenotypic characteristics, including groups characterized by large fruits and thick pericarps and ornamental groups characterized by dense flowering and large flowers. Flow cytometry classified the 30 selected accessions as decaploid, and resequencing showed that most variants occurred in non-coding regions. Genome-wide association analysis identified 9 significant SNPs (−log10(P) > 8.63) associated with petal number, flower diameter, locule number, and flower density (3, 1, 3, and 2 SNPs, respectively), including a flower-diameter signal on GWHERBT00000039 and signals on GWHERBT00000008 and GWHERBT00000036. These results integrate phenotypic and genomic evidence for *C. hainanica* and provide germplasm resources and preliminary candidate regions for further validation and ornamental improvement alongside fruit-trait evaluation.

## 1. Introduction

*Camellia oleifera* is one of the world’s major woody oil crops and is valued for its nutritional, economic, and ecological importance. China is the center of origin and the principal production region for oil-tea camellias [1]. Among the diverse taxa in this group, *Camellia hainanica* is endemic to Hainan Island. Long-term geographic isolation, a warm and humid tropical climate, and natural and anthropogenic selection have contributed to its broad ecological adaptability and distinctive germplasm diversity [2,3]. The seeds yield high-quality edible oil, while variation in floral and plant architecture also confers potential ornamental value.

Systematic surveys and germplasm conservation have supported the selection and utilization of elite *C. hainanica* resources [4,5]. Studies of the physicochemical properties and fatty acid composition of *C. hainanica* seed oil have provided a basis for evaluating its economic potential [6]. Taxonomic and genomic evidence supports *C. hainanica* as a distinct species and indicates the occurrence of octoploid and decaploid cytotypes in tropical oil-tea camellia populations [7,8]. Compared with *C. oleifera*, *C. hainanica* exhibits distinctive floral, fruit, growth, and oil-quality characteristics.

Phenotypic characterization remains fundamental to germplasm evaluation because it captures morphological and physiological variation relevant to adaptation, classification, and breeding [9,10,11,12,13]. Although phenotypic traits are influenced by both genotype and environment, quantitative and qualitative trait analyses can reveal population differentiation, identify potentially useful descriptors, and prioritize accessions for cultivar development.

Whole-genome resequencing enables the detection of single-nucleotide polymorphisms (SNPs), insertions/deletions (InDels), structural variants, and copy-number variation at high resolution [14]. Genome-wide association studies (GWASs) integrate genomic and phenotypic data to identify loci associated with agronomic, quality, resistance, and ornamental traits [15]. Extreme-phenotype or multi-trait selection strategies can increase the efficiency of detecting loci underlying complex variations [16].

In this study, 31 phenotypic traits were evaluated in 100 *C. hainanica* accessions using descriptive statistics, diversity indices, cluster analysis, and correlation analysis. With ornamental improvement as the main objective, flower-diameter variation guided the selection of 30 accessions for whole-genome resequencing and GWASs. The aim was to identify genomic regions associated with floral traits for future candidate-gene research, while maintaining fruit and seed characterization within the broader germplasm evaluation.

## 2. Results

### 2.1. Variation in Quantitative Traits

The 22 quantitative traits measured across 100 *C. hainanica* accessions showed broad variation (Table 1). CV values ranged from 8.81% to 56.85%. Flower density had the highest CV (56.85%), indicating substantial variation in this trait, whereas fruit shape index had the lowest CV (8.81%), indicating comparatively uniform fruit proportions. Ten-grade H′ values ranged from 1.72 to 2.08, with the highest values for leaf length, seed number, and fresh seed weight.

Among fruit-related traits, single-fruit weight showed substantial variation (CV = 28.36%), whereas fruit length and fruit diameter had CV values of 10.24% and 10.60%, respectively. Floral traits showed moderate-to-high variation: CV values were 18.06% for petal number, 12.15% for flower diameter, 12.55% for petal length, 17.46% for petal width, and 23.65% for petal-tip split depth. Leaf length, leaf width, and leaf area had CV values of 10.99%, 13.35%, and 20.46%, respectively.

### 2.2. Variation in Qualitative Traits

Nine qualitative traits were evaluated (Table 2). Category frequencies varied among accessions. Leaf shape, leaf margin, and fruit color had the highest diversity indices, whereas flower color was comparatively uniform.

Yellow and yellowish-brown fruits accounted for 83% of the accessions, and spherical or tangerine-shaped fruits accounted for 92%. Conical kernels represented 70% of the accessions, and dark-brown seed coats represented 75%. Single and semi-double flowers accounted for 76% of the accessions, while 91% had pure-white flowers. Green or dark-green leaf surfaces represented 96% of the accessions, and elliptic or obovate leaves were the predominant leaf shape categories.

### 2.3. Phenotypic Distribution and Selection of Accessions

**Figure 1:**
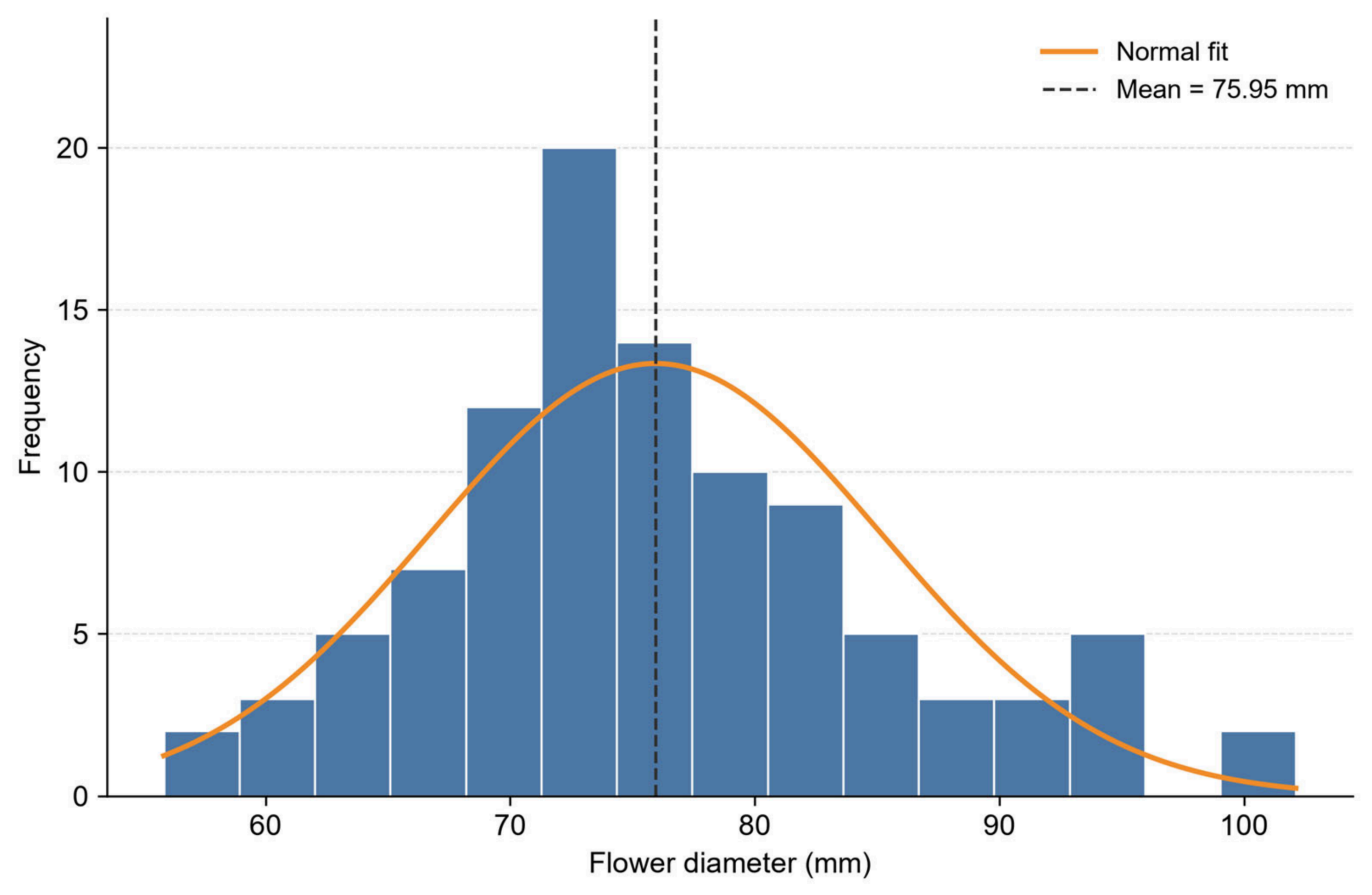
Frequency distribution of flower diameter in 100 *C. hainanica* accessions. The dashed line indicates the mean (75.95 mm); the solid curve is a normal fit to the original observations.

Flower diameter ranged from 55.84 to 102.12 mm in the 100 accessions, with a mean of 75.95 mm (Figure 1). This distribution provided a phenotypic basis for selecting materials with contrasting and intermediate flower sizes for ornamental evaluation. The 30 selected accessions spanned the same flower-diameter range, with a mean of 75.68 mm and SD of 12.72 mm, compared with a mean of 75.95 mm and an SD of 9.23 mm in the full collection (Table S2; Section 4.4).

### 2.4. Cluster Analysis

**Figure 2:**
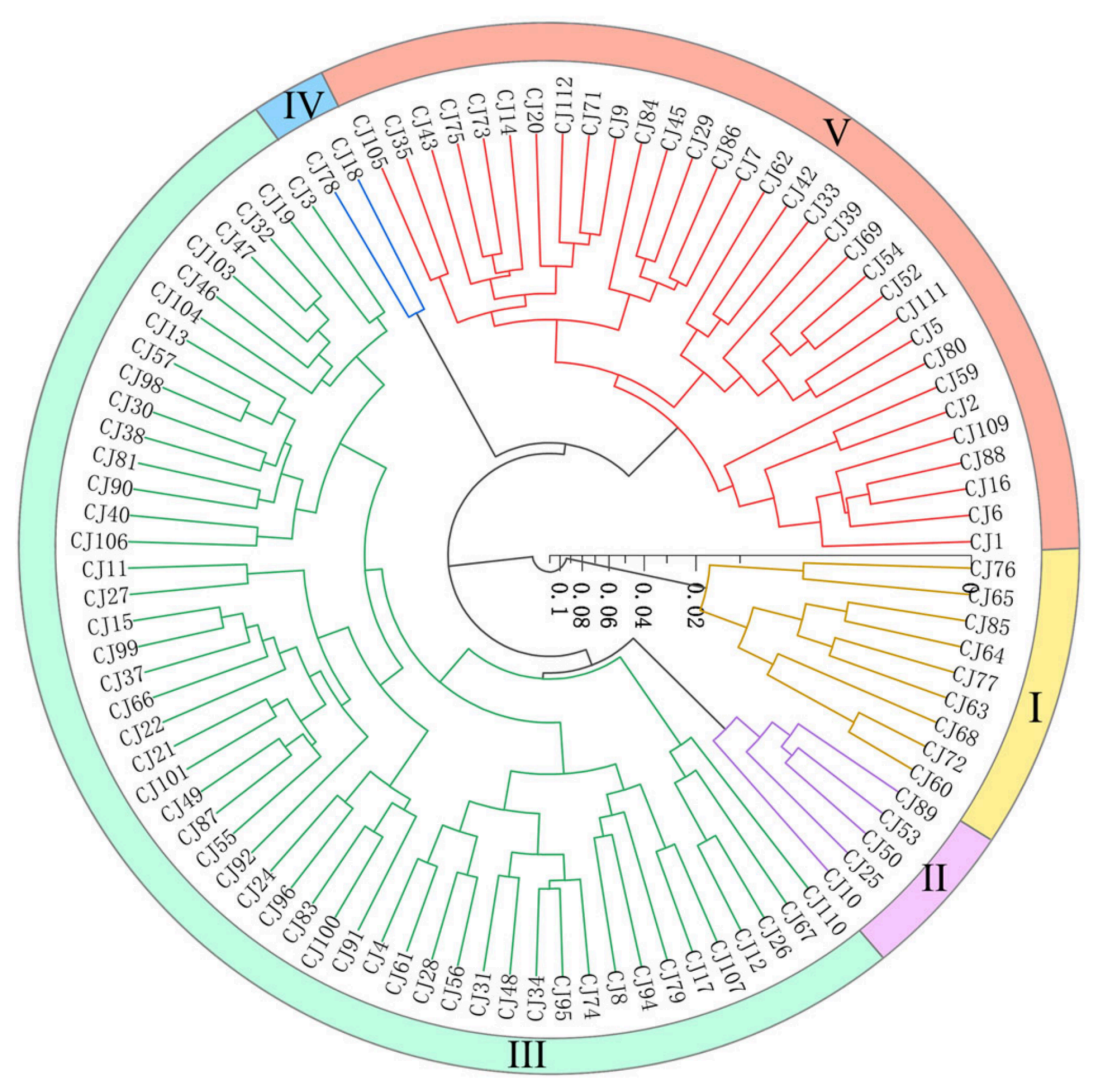
Hierarchical clustering of 100 *C. hainanica* accessions into five phenotypic groups.

Hierarchical cluster analysis classified the 100 accessions into five groups (Figure 2). Cluster I was characterized by dark-colored, rounded fruits, relatively high locule counts, and vigorous growth. Cluster II contained accessions with oblate fruits, thick pericarps, and shorter stature. Cluster III, the largest group, was characterized by heavier and more elongated fruits, high flower density, and compact growth. Cluster IV had lighter-colored, rounded fruits and taller trees, whereas Cluster V combined elongated fruits, thick pericarps, and open crowns.

The clusters represented contrasting phenotypic characteristics. Clusters I and III contained accessions with favorable fruit and seed characteristics; CJ72 and CJ92 were notable for locule number and fruit weight, respectively. Clusters II and V contained accessions with ornamental potential, including CJ10 for flower diameter and CJ80 for flower density. These accessions represent candidate materials for further replicated evaluation and breeding.

### 2.5. Correlation Analysis

**Figure 3:**
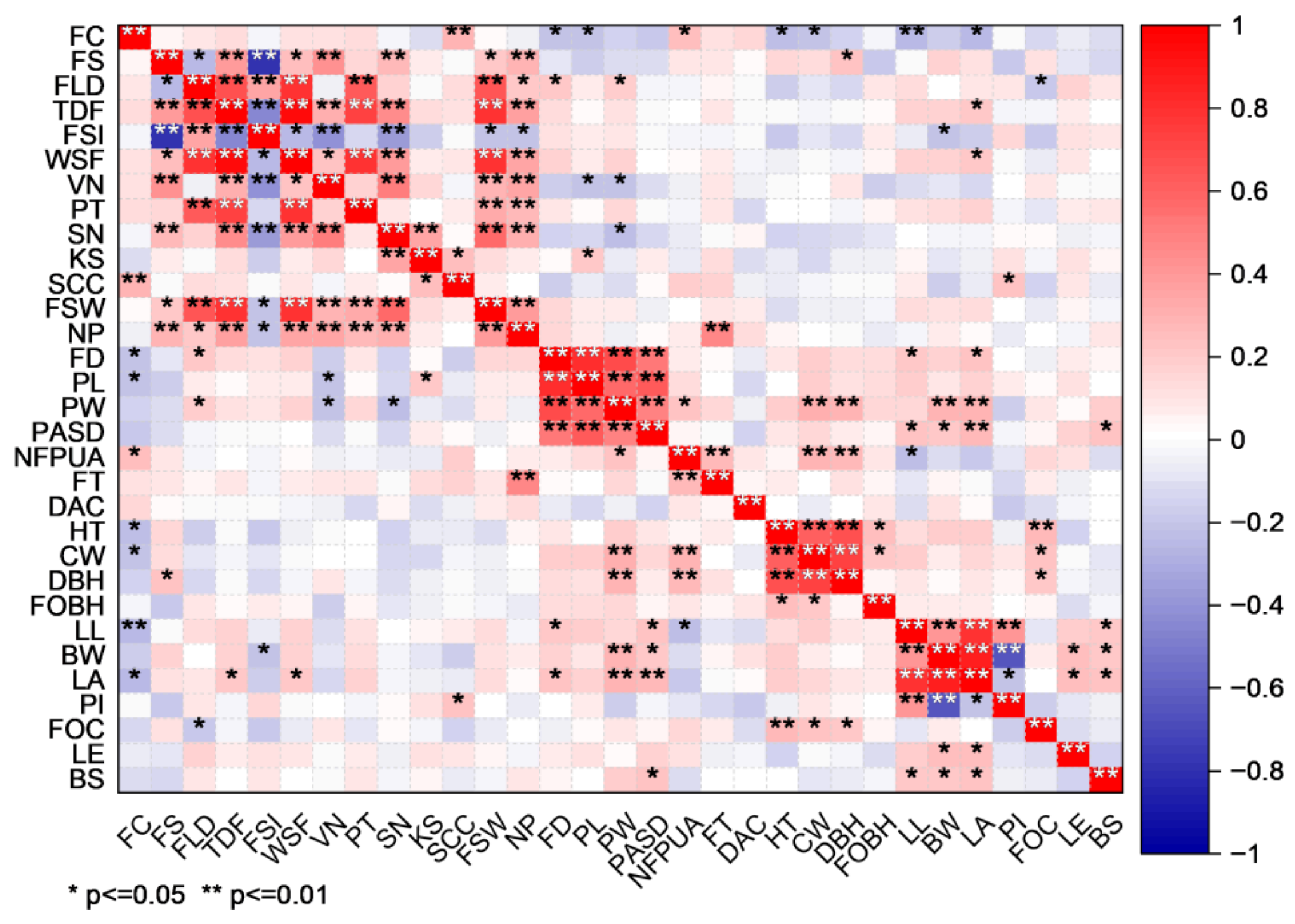
Correlation matrix of phenotypic traits in 100 *C. hainanica* accessions. * *p* < 0.05; ** *p* < 0.01.

Correlation analysis identified significant relationships among many phenotypic traits (Figure 3). Fruit dimensions were strongly and positively correlated with fruit weight (r = 0.782–0.952), and leaf area was strongly correlated with leaf width. Crown width and diameter at breast height were also positively associated (r = 0.739). The fruit shape index was negatively correlated with transverse fruit dimensions or categorical fruit shape (r = −0.799 to −0.453).

### 2.6. Whole-Genome Resequencing Results

#### 2.6.1. Sequencing Data Quality

Whole-genome resequencing was performed for 30 selected accessions. Flow cytometry classified all 30 selected samples as decaploid. The total sequencing yield was 2820.44 Gb (Table 3). Across samples, Q20 and Q30 values averaged 98.10% and 93.38%, respectively, and mean GC content was 38.52%. Base yield per sample ranged from 90.24 to 111.33 Gb, and GC content ranged from 38.24% to 39.28%, indicating consistent sequencing quality for downstream variant analysis.

#### 2.6.2. SNP and InDel Variation

Variant calling identified 236,808,965 unique SNPs and 12,914,933 unique InDels. Region-based annotation produced 265,985,527 SNP effects and 15,266,062 InDel effects (Table 4). Most annotation effects were intergenic (84.705% of SNP effects and 78.285% of InDel effects). Exonic effects represented 0.817% and 0.517%, whereas intronic effects represented 3.344% and 5.419%, respectively. Upstream and downstream regions together accounted for 10.943% of SNP effects and 15.356% of InDel effects; UTR effects accounted for 0.154% and 0.359%, respectively.

The base-change matrix comprised 236,808,965 unique SNP sites, including 178,997,017 transitions and 57,811,948 transversions (site-level Ts/Tv = 3.0962; Table 5). SnpEff additionally reported a raw observed-event Ts/Tv ratio of 4.2225, based on 5,061,233,502 transitions and 1,198,626,205 transversions. These statistics use different counting levels.

Predicted annotation effects were classified into four impact categories: high, moderate, low, and modifier (Table 6). Modifier effects predominated, accounting for 99.14% of SNP annotation effects and 99.42% of InDel annotation effects, and mainly arose from non-coding regions. High-impact effects were rare, accounting for 0.04% of SNP effects and 0.38% of InDel effects. Moderate-impact effects, including coding-sequence changes, accounted for 0.50% of SNP effects and 0.14% of InDel effects; low-impact effects accounted for 0.33% and 0.05%, respectively.

### 2.7. Genome-Wide Association Analysis

After filtering, 21,278,301 SNPs were retained for association analysis.

**Figure 4:**
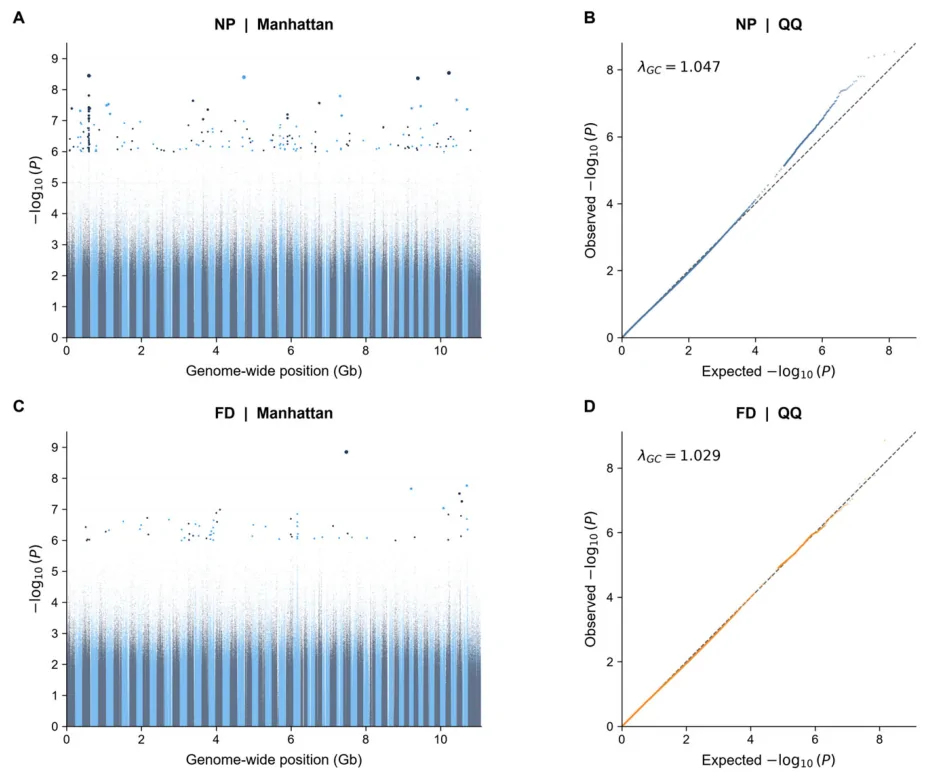
GWAS results for petal number (NP) and flower diameter (FD). (**A**) Manhattan plot for NP; (**B**) QQ plot for NP (λGC = 1.047); (**C**) Manhattan plot for FD; (**D**) QQ plot for FD (λGC = 1.029). Significant SNPs had −log10(P) > 8.63.

A total of 9 significant SNPs with −log10(P) > 8.63 were detected across the four traits. GWASs for petal number identified 3 significant SNPs, with a prominent association on GWHERBT00000008 (Figure 4). The strongest signal was located at 74,402,962 bp. Additional signals were detected at 182,770,806 bp on GWHERBT00000021 and 177,291,111 bp on GWHERBT00000003. One significant SNP for flower diameter was detected at 81,443,240 bp on GWHERBT00000039.

**Figure 5:**
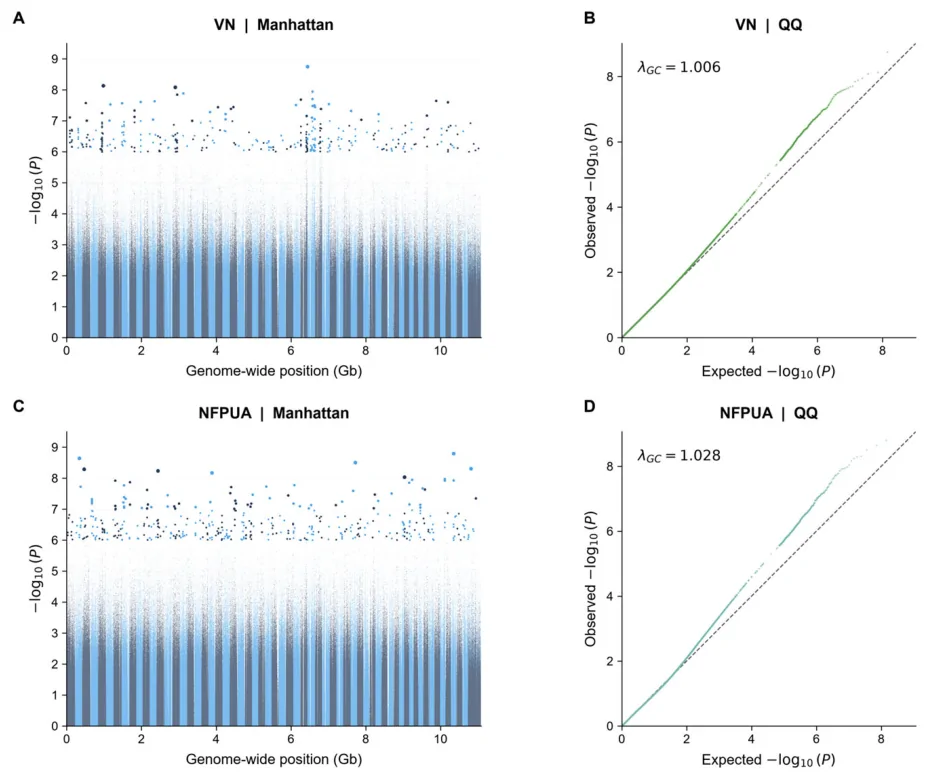
GWAS results for locule number (VN) and flower density per unit area (NFPUA). (**A**) Manhattan plot for VN; (**B**) QQ plot for VN (λGC = 1.006); (**C**) Manhattan plot for NFPUA; (**D**) QQ plot for NFPUA (λGC = 1.028). Significant SNPs had −log10(P) > 8.63.

Flower density was associated with 2 significant SNPs, with the strongest signal at 76,638,829 bp on GWHERBT00000058 and an additional signal at 120,330,864 bp on GWHERBT00000002 (Figure 5). Three significant SNPs were detected for locule number, including the strongest signal at 181,338,542 bp on GWHERBT00000036 and additional signals at 3,861,510 bp on GWHERBT00000034 and 55,383,490 bp on GWHERBT00000020. Together, these results suggest that multiple loci may contribute to variation in petal number, locule number, and flower density, whereas flower diameter showed a single significant association in this panel.

Allele frequencies, additive effects, standard errors (SEs), and 95% confidence intervals (CIs) of the nine reported SNPs are provided in Table S4. MAF ranged from 0.0690 to 0.4042. The FD-associated ALT allele had an estimated effect of +3.937732 mm per copy (SE = 0.423862; 95% CI, 3.064771 to 4.810693). NP effects included both positive and negative estimates, whereas the reported NFPUA and VN effects were positive. All nine model-based 95% CIs excluded zero. Effect directions were unchanged across 261 leave-one-accession-out fits and were consistent in complete-case and mean-imputation analyses.

QQ plots broadly followed the expected distribution, with upper-tail deviations more apparent for NP, VN, and NFPUA than for FD (Figure 4 and Figure 5). The genomic inflation factors (λGC; NP, 1.047; FD, 1.029; VN, 1.006; NFPUA, 1.028) were close to 1, indicating limited overall inflation of the test statistics.

**Figure 6:**
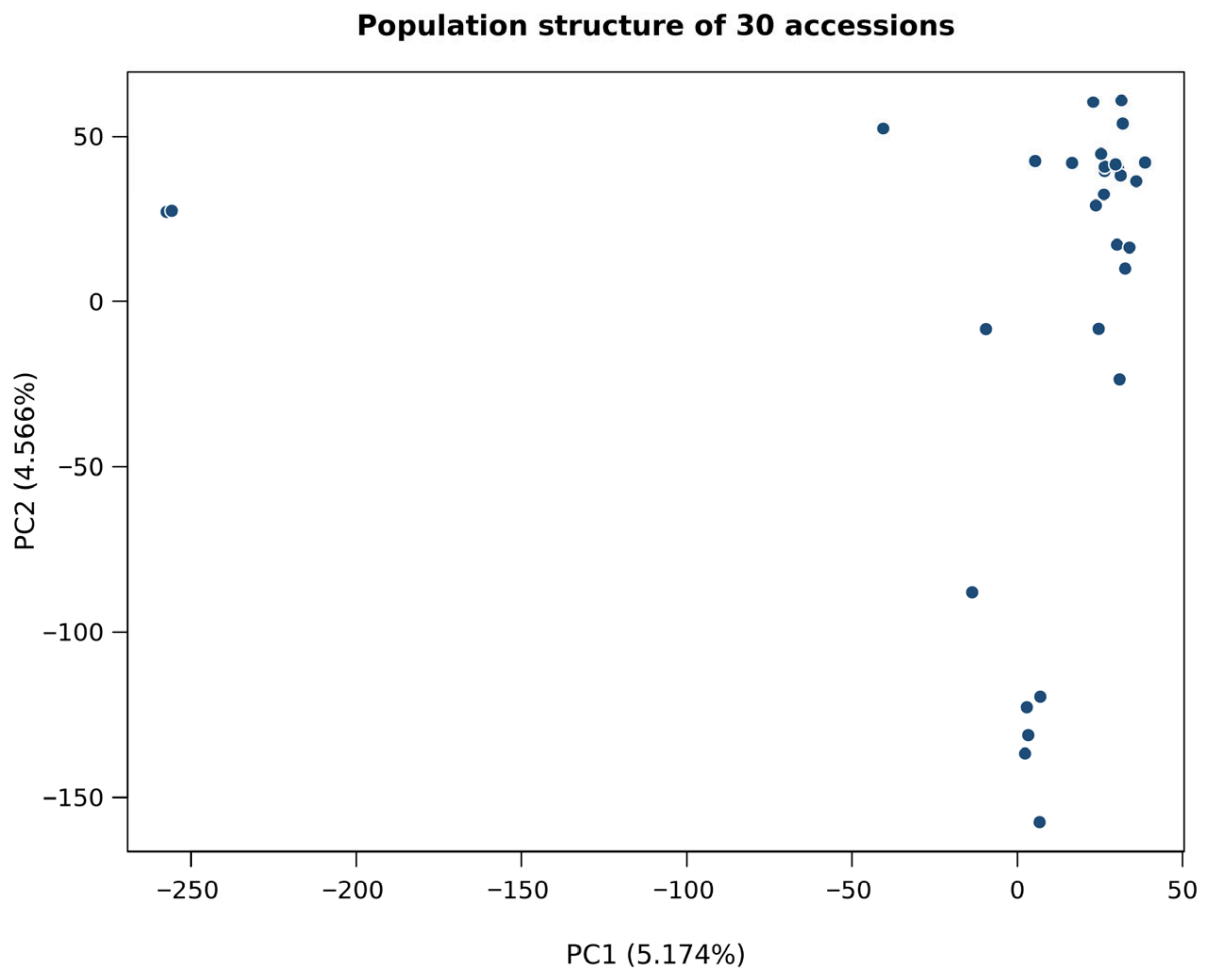
PC1–PC2 plot of the 30 resequenced accessions.

**Figure 7:**
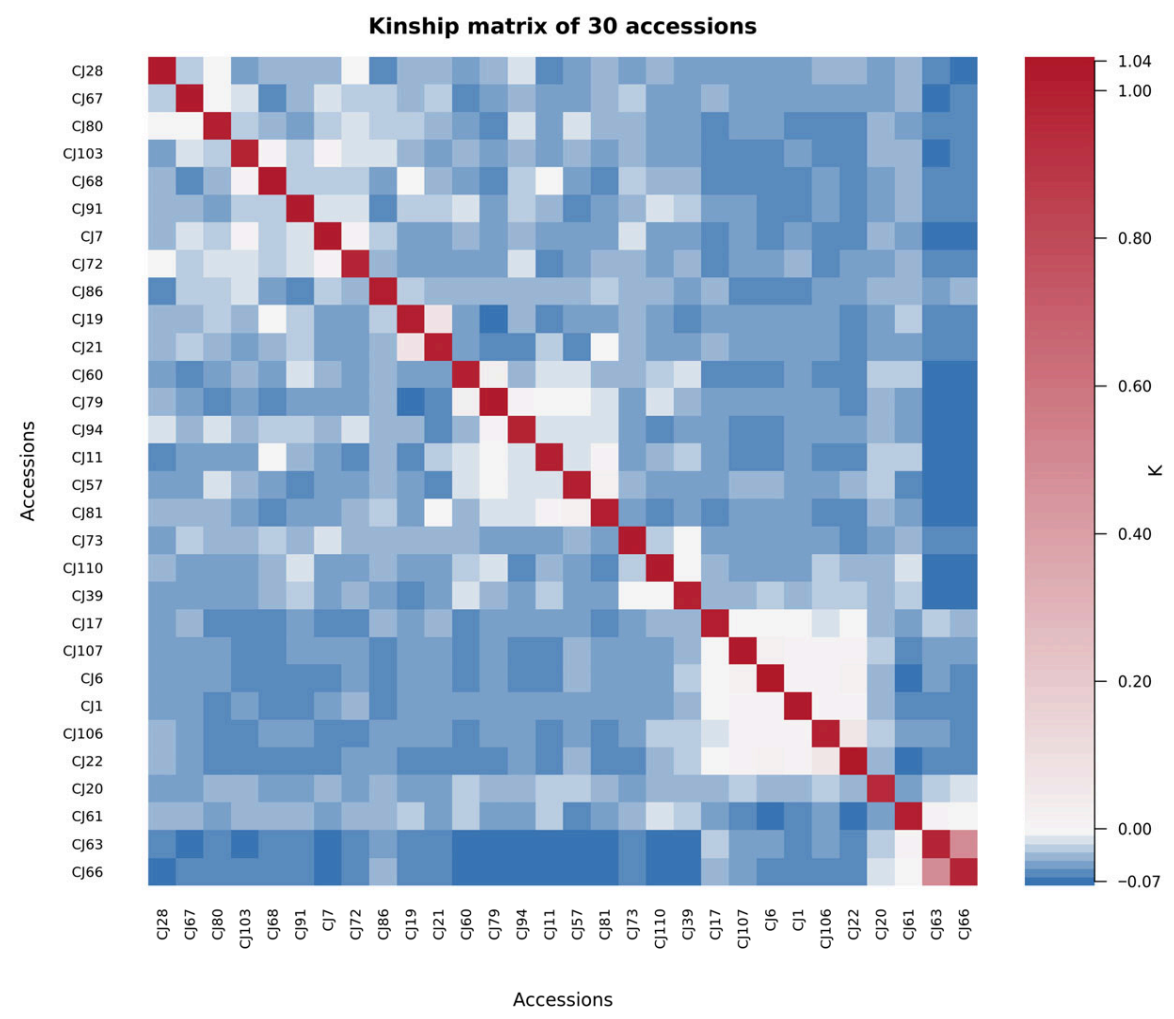
Heatmap of the kinship matrix for 30 accessions.

PCA of all 30 resequenced accessions showed that PC1 and PC2 explained 5.174% and 4.566% of marker variation (Figure 6). Several accessions were separated from the main cluster, suggesting heterogeneity within the panel. The kinship heatmap showed mostly small off-diagonal values and some higher pairwise values, indicating variation in genomic relatedness (Figure 7).

**Figure 8:**
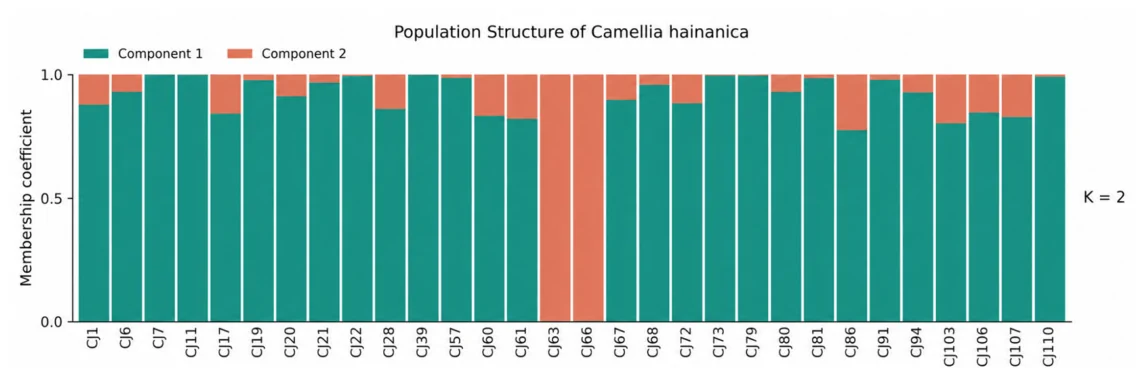
STRUCTURE analysis of the 30 resequenced *Camellia hainanica* accessions.

STRUCTURE analysis showed a pronounced ΔK peak at K = 2 (ΔK = 64.05), with consistent membership estimates across five runs (Figure 8). A small subset of accessions differed from the majority in their inferred genetic components. Partitions at higher K values were less consistent across runs, although likelihood values continued to increase.

Gene annotations for the nine significant SNP regions are summarized in Table S3. These included a Ras-superfamily small GTPase for the NP-associated region on GWHERBT00000008, a SWEET-family sugar transporter for the NFPUA-associated region on GWHERBT00000058, and a NOT2/3/5-family protein for the VN-associated region on GWHERBT00000036. The FD-associated region was represented by the agglutinin-like protein YC.10G0008000-2P.

## 3. Discussion

Evaluation of 31 traits across 100 *C. hainanica* accessions revealed extensive phenotypic variation, particularly in flower density, single-fruit weight, fresh seed weight, and branching traits. For ornamental evaluation, flower diameter and petal number describe flower size and form, whereas flower density reflects flowering within a defined canopy area. As in multi-trait evaluations of *C. oleifera* germplasm [9], these differences provide a basis for identifying accessions with complementary characteristics, with particular emphasis here on floral variation in *C. hainanica*. The comparatively low CV of fruit shape index indicates more uniform fruit proportions rather than absolute fruit size. Together, these patterns support the evaluation of flower size, floral form, and flowering density alongside fruit characteristics when selecting materials for different breeding objectives.

Cluster analysis identified five groups with contrasting breeding potential. Clusters I and III included accessions with favorable fruit and seed characteristics, whereas Clusters II and V contained accessions with ornamental potential such as large flowers or high flower density. CJ10 and CJ80 may be prioritized for evaluating flower diameter and density, respectively. Accessions in Cluster III showed relatively high single-fruit weight, while CJ72 and CJ92 were notable for locule number and fruit weight, respectively. Accessions with these fruit characteristics may serve as reference materials for subsequent screening for yield potential. The positive correlations between fruit dimensions and weight also support their joint use in fruit-size evaluation. Previous evaluations of *C. oleifera* have considered fruit characteristics together with seed traits and yield measurements [17], providing a direction for further assessment of these materials. Because yield per tree, seed oil content, and oil yield were not measured here, their actual yield and oil-production performance remain to be evaluated.

Whole-genome resequencing of 30 phenotype-selected accessions generated high-quality data. The high Q20 and Q30 values and narrow GC-content range indicate consistent sequencing quality. The site-level Ts/Tv ratio was 3.10, whereas the SnpEff raw observed-event ratio was 4.22. Both values indicate an excess of transitions and summarize substitution patterns at the site and observed-event levels, respectively.

Most SNP and InDel annotation effects were assigned to non-coding regions, and modifier effects comprised the largest functional class. These annotations describe the genomic distribution and predicted impacts of the detected variants, providing a resource for further investigation of *C. hainanica* diversity. The gene annotations in Table S3 provide a basis for prioritizing gene-expression analysis during flower development and subsequent functional testing. The less frequent high- and moderate-impact effects may also be examined in future functional analyses.

This study identifies 9 significant SNPs with −log10(P) > 8.63 associated with floral and fruit traits. The flower-diameter signal on GWHERBT00000039 at 81,443,240 bp directly addresses the associations with flower-size variation. Signals on GWHERBT00000008 for petal number and GWHERBT00000036 for locule number provide further candidate regions for gene identification. In *C. japonica*, altered *CjAG* expression is associated with double-flower development, and ectopic expression in *Arabidopsis* changes petal and stamen development [18]. This work illustrates the value of examining floral-development pathways when investigating ornamental variation in *Camellia*. The flower-diameter, petal-number, and flower-density associations in the present study provide complementary starting points for studying flower size, floral form, and flowering display. The Ras-superfamily, SWEET, and NOT2/3/5 annotations (Table S3) provide initial targets for expression and functional studies. Together with the phenotypic evaluation, these findings provide germplasm resources and preliminary candidate regions for further research.

These associations should be interpreted in the context of the study design. Flower-diameter-guided sampling focused the analysis on ornamental value variation, although the small, non-random panel may affect detection power, effect estimates, and their generalizability. Tree age and variation in soil microbial and environmental conditions may contribute to the observed phenotypes, and correlations involving derived indices may partly reflect the shared measurements. Use of a related tetraploid as reference also warrants attention in subsequent validation. Including principal components and kinship in the model helps account for population structure and relatedness but does not eliminate all sampling and environmental effects. STRUCTURE provided additional evidence of genetic differentiation within the panel. The inferred genetic components are not equivalent to the five phenotypic groups and remain exploratory given the small sample size and variable genotype completeness. Repeated phenotyping and validation in larger independent populations are needed before marker-assisted use. The effect estimates in Table S4 describe the direction and magnitude of associations within this panel; they do not establish causal effects or predict breeding responses in other populations. Validation in this study was limited to internal robustness checks. The associated loci still require confirmation in larger independent populations.

## 4. Materials and Methods

### 4.1. Plant Materials and Experimental Site

Plant materials were collected from a Camellia seedling plantation at Jinbo State Farm, Changjiang County, Hainan Province, China (19.276876° N, 109.126088° E; 351.87 m above sea level). The site is located in the central mountainous region of Hainan Island and has a tropical monsoon climate. The plantation was established in the 1960s using seeds collected across Hainan Island and contains trees approximately 20–70 years old. One hundred healthy seed-grown individuals, estimated to be 30–40 years old, were selected. The trees grew under natural conditions, with manual fruit harvesting at maturity as the only management intervention.

### 4.2. Phenotypic Trait Measurement

For each accession, ten healthy mature leaves, ten fully opened flowers, and ten pest-free mature fruits were randomly sampled from the outer canopy at appropriate developmental stages. Measurements were performed in the field or laboratory following standard botanical procedures [19].

#### Morphological Measurements

Leaf length, leaf width, flower diameter, petal length, petal width, fruit length, fruit diameter, and pericarp thickness were measured with a vernier caliper (precision: 0.01 mm). Fruit and fresh seed weights were measured with an electronic balance (precision: 0.01 g), and petal, seed, and locule numbers were counted directly. Flower density (NFPUA) was calculated as the mean number of flowers recorded in three 0.5 m × 0.5 m canopy quadrats per tree, expressed as flowers per 0.25 m^2^. Derived indices were calculated as follows: leaf area = 2/3 × leaf length × leaf width; leaf shape index = leaf length/leaf width; and fruit shape index = fruit length/fruit diameter. Nine qualitative traits—fruit color, fruit shape, seed-kernel shape, seed-coat color, flower type, flower color, leaf-surface color, leaf-margin type, and leaf shape—were evaluated according to LY/T 2742-2016 and published classification criteria [20]. Trunk circumference was measured in mm; DBH (cm) = circumference/(10 × π). Tree height, crown width, and first-branch height were recorded in the field. Traits and measurement procedures are listed in Table 1, Table 2 and Table S1; NP, FD, VN, and NFPUA were analyzed by GWASs, and the remaining traits were used for phenotypic characterization.

### 4.3. Statistical Analysis of Phenotypic Traits

Measurements were averaged within each accession. Quantitative traits were summarized using the mean, range, standard deviation (SD), coefficient of variation (CV), and Shannon–Wiener diversity index (H′). Qualitative traits were summarized by category frequencies. For quantitative traits, values were assigned to ten grades based on the overall mean ($\overset{-}{x}$) and sample SD calculated using n − 1: grade 1 represented values < $\overset{-}{x}$ − 2 SD, grade 10 represented values ≥ $\overset{-}{x}$ + 2 SD, and intermediate grades were separated by 0.5 SD [21]. The diversity index was calculated as H′ = −∑p_i_ln(p_i_), where p_i_ is the relative frequency of grade or category i. Cluster and correlation analyses were conducted using standardized phenotypic data; two-tailed *p* values < 0.05 were considered statistically significant. Statistics were calculated across 100 accession-level values; CV (%) = SD/mean × 100, with mean and SD in the same unit. Correlations involving categorical codes were interpreted descriptively.

### 4.4. Phenotype-Informed Selection of Accessions for Association Analysis

Flower diameter was the focal trait for selecting materials to investigate ornamental trait variation. The distribution in Figure 1 was used to visualize flower-size variation and select 30 accessions with contrasting and intermediate phenotypes, drawing on extreme-phenotype sampling principles [22]. Field, phenotype, and sequencing identifiers corresponded directly; CJ codes identify individual seed-grown trees (Table S2). GWASs were performed on individual accessions, not on pooled samples. NP and NFPUA were included to represent floral form and display, while VN extended the analysis to fruit structure.

### 4.5. Whole-Genome Resequencing, Variant Calling, and GWASs

Raw FASTQ reads were processed with fastp [23] and aligned to the chromosome-level tetraploid *C. oleifera* assembly (Genome Warehouse accession GWHERBT00000000) [24] using BWA-MEM in the Sentieon workflow [25,26]. After duplicate removal, per-sample genomic variant call format files were generated with Sentieon Haplotyper and jointly genotyped using GATK CombineGVCFs and GenotypeGVCFs [27]. Biallelic SNPs were extracted from the jointly genotyped data of the 30 accessions, and hard filtering retained 231,135,138 sites. Allele frequencies and missingness were then recalculated using the actual decaploid genotypes. Sites with MAF ≥ 0.01 and missingness ≤ 0.10 were retained, yielding 98,671,074 candidate SNPs. A custom decaploid dosage-based LD-pruning program was applied using a window of 50 markers and a step of 10 markers; redundant markers were removed when r^2^ > 0.20, leaving 21,286,114 SNPs. Variant tags were recalculated, and filtering was repeated, resulting in 21,278,301 SNPs for association analysis. A fixed set of 100,000 eligible markers was used for structuring assessment, with marker-mean imputation of missing dosages. PCA for Figure 6 used standardized dosages at these markers for all 30 resequenced accessions. The PC covariates and kinship matrix (K) used in GWASs were estimated for the association panel. K was calculated from marker-centered cross-products normalized to a mean diagonal of 1. Figure 7 presents a kinship matrix recalculated for all 30 resequenced accessions using the same marker panel and procedure.

STRUCTURE 2.3.4 was run at ploidy 10 using 1000 SNPs selected from the structure marker panel after MAF ≥ 0.05, site missingness ≤ 0.10 and dosage-based LD filtering (50-marker window, one-marker step, r^2^ threshold of 0.20). The admixture model with correlated allele frequencies was evaluated for K = 1–6, with five runs per K, a burn-in of 10,000 iterations, and 20,000 subsequent MCMC iterations. Missing calls were retained, and LnP(D), ΔK and replicate consistency were examined.

GWASpoly used an additive model with ALT dosages of 0–10, PC1 and PC2 as fixed covariates, and K to model random polygenic effects (P3D = TRUE; LOCO = FALSE) [28,29]. Missing GWAS dosages were imputed using the population mode. SNPs with −log10(P) > 8.63 were considered significant. λGC was calculated by dividing the median χ^2^ statistic with 1 degree of freedom by 0.454936; the median was approximated from a histogram of −log10(P) values with a bin width of 0.0001. Ploidy of all 30 accessions was determined by leaf-nucleus flow cytometry against a diploid *C. chekiangoleosa* standard, following Zhang et al. [30].

For Table S4, ALT allele frequency was calculated from observed decaploid dosages as the total ALT copy number divided by ten times the number of non-missing genotypes. MAF was the smaller of the REF and ALT frequencies. Additive effects were taken from the corresponding GWASpoly effect outputs and expressed per additional ALT copy. These precise and sensitive calculations reproduced the archived GWASpoly fit for 30 accessions. SEs were obtained from the coefficient covariance matrix, and 95% CIs used the t distribution with the corresponding residual degrees of freedom. Internal sensitivity analyses compared mode imputation with the mean imputation and the complete-case fits and repeated each model after omitting one accession. Original PC values and K submatrices were retained, and null-model variance components were re-estimated for each subset.

Significant SNP-associated regions were annotated by relating SNP coordinates to reference gene models and comparing the corresponding protein sequences against the Pfam database. Table S3 summarizes reference gene models, their distances from the associated SNPs, and protein annotations.

## 5. Conclusions

By integrating phenotypic evaluation with whole-genome resequencing and GWASs, this study characterized diversity in decaploid *C. hainanica* with an emphasis on ornamental floral traits. The 100 accessions showed variation across 31 traits and were classified into five phenotypic groups, providing materials with contrasting floral and fruit characteristics. Flow cytometry classified the 30 selected accessions as decaploid, and most annotated variants occurred in non-coding regions. PCA and STRUCTURE analyses suggested genetic differentiation within the selected panel. GWASs identified 9 significant SNPs with −log10(P) > 8.63 associated with petal number, flower diameter, locule number, and flower density, including signals on GWHERBT00000039 for flower diameter, GWHERBT00000008 for petal number, and GWHERBT00000036 for locule number. Internal checks supported consistent effect directions within this panel. These results provide germplasm resources and preliminary candidate regions for investigating flower size, floral form, and flowering density, alongside fruit and seed evaluation. Accessions with contrasting floral characteristics may support ornamental improvement, while those traits with favorable fruit characteristics provide reference materials for subsequent screening for yield potential. Further phenotypic evaluation and validation of the associated loci in larger independent populations are required before a marker-assisted breeding scheme can be used.

## Figures and Tables

**Table 1:** Descriptive statistics and diversity indices for quantitative traits in 100 *C. hainanica* accessions.

Traits	Max	Min	Range	Mean	SD	CV (%)	H′
Fruit Length (FLD, cm)	5.512	3.342	2.17	4.3307	0.4435	10.24	2.04
Fruit Diameter (TDF, cm)	6.723	3.492	3.231	5.1010	0.5409	10.60	2.03
Fruit Shape Index (FSI)	1.04	0.7	0.34	0.8530	0.07519	8.81	1.96
Single-Fruit Weight (WSF, g)	115.68	28.4	87.28	63.3050	17.9533	28.36	1.91
Locule Number (VN)	4.7	2.9	1.8	3.5089	0.4541	12.94	1.93
Pericarp Thickness (PT, cm)	0.9547	0.415	0.5397	0.5979	0.1147	19.18	1.93
Seed Number (SN)	10.4	1.56	8.84	6.2115	1.7854	28.74	2.08
Fresh Seed Weight (FSW, g)	34.85	9.87	24.98	22.0313	5.7348	26.03	2.08
Petal Number (NP)	8.5	5	3.5	6.7072	1.2111	18.06	1.72
Flower Diameter (FD, cm)	10.212	5.584	4.628	7.5945	0.9226	12.15	2.04
Petal Length (PL, cm)	5.179	2.65	2.529	3.7549	0.4711	12.55	1.93
Petal Width (PW, cm)	3.506	1.409	2.097	2.3759	0.4149	17.46	2.04
Petal-Tip Split Depth (PASD, cm)	1.2957	0.262	1.0337	0.7701	0.1821	23.65	2.05
Flower Density (NFPUA, flowers per 0.25 m^2^)	31.33	1	30.33	11.0176	6.2631	56.85	1.95
Tree Height (HT, m)	9.6	3.1	6.5	5.7910	1.0950	18.91	2.01
Crown Width (CW, m)	7.3	2.3	5	4.2130	0.9371	22.24	1.94
Diameter at Breast Height (DBH, cm)	52.5211	9.5493	42.9718	27.0627	9.4278	34.84	2.00
First-Branch Height (FOBH, cm)	64	2	62	22.5020	12.4279	55.23	2.02
Leaf Length (LL, cm)	8.357	5.146	3.211	6.7574	0.7424	10.99	2.08
Leaf Width (BW, cm)	4.427	2.065	2.362	3.1723	0.4236	13.35	2.04
Leaf Area (LA, cm^2^)	24.6643	8.739	15.9253	14.3746	2.9407	20.46	2.02
Leaf Shape Index (PI)	3.11	1.59	1.52	2.1557	0.2954	13.70	2.00

Note: Statistics use 100 accession-level values and sample SD. Mean and SD share the same unit; CV was calculated before rounding. H′ uses natural logarithms and ten SD-based grades.

**Table 2:** Frequency distributions and diversity indices for qualitative traits in 100 *C. hainanica* accessions.

Traits	Category Frequency (%)	H′
Fruit Color (FC)	9/38/45/8	1.15
	Green/Yellow/Yellowish brown/Brown	
Fruit Shape (FS)	2/6/24/68	0.85
	Olive-shaped/Ovoid/Spherical/Tangerine-shaped	
Kernel Shape (KS)	1/8/70/21	0.83
	Spherical/Hemispherical/Conical/Kidney-shaped	
Seed Coat Color (SCC)	4/11/75/10	0.82
	Brown/Brownish/Dark brown/Black	
Flower Type (FT)	44/32/24	1.07
	Single/Semi-double/Double	
Flower Color (DAC)	9/91	0.30
	White with red spots/Pure white	
Leaf Surface Color (FOC)	4/33/63	0.79
	Yellow-green/Green/Dark green	
Leaf Margin (LE)	7/26/38/29	1.26
	Fine serration/Medium serration/Coarse serration/Heavy serration	
Leaf Shape (BS)	10/51/3/20/16	1.29
	Lanceolate/Elliptic/Ovate/Obovate/Narrow obovate	

**Table 3:** Whole-genome resequencing data quality for 30 *C. hainanica* accessions.

No.	Base Yield (Gb)	Q20 (%)	Q30 (%)	GC (%)
CJ103	95.86	98.28	94.03	38.48
CJ106	111.33	98.41	94.54	38.31
CJ107	92.58	98.34	94.31	38.33
CJ110	93.38	98.49	94.7	38.38
CJ11	92.89	98.06	92.85	38.42
CJ17	94.08	98.01	92.8	38.41
CJ19	90.36	98.05	92.85	38.51
CJ1	91.35	98.08	92.87	38.69
CJ20	91.19	98.13	93.04	39.28
CJ21	93.58	97.73	92.44	38.24
CJ22	92.1	98.03	92.84	38.43
CJ28	90.93	97.88	92.74	38.93
CJ39	98.2	97.8	92.52	38.48
CJ57	95	97.98	93.09	38.66
CJ60	97.39	98.04	93.22	38.76
CJ61	94.4	97.92	92.96	38.51
CJ63	94.99	97.83	92.66	38.4
CJ66	93.87	97.94	92.97	38.35
CJ67	90.35	98.1	93.53	38.28
CJ68	92.96	98.07	93.46	39
CJ6	90.95	98.03	92.78	38.31
CJ72	103.32	98	93.29	38.7
CJ73	92.03	98.13	93.62	38.47
CJ79	90.45	98.26	94.1	38.38
CJ7	91.81	98.15	93.11	38.55
CJ80	93.59	97.74	92.39	38.49
CJ81	90.24	98.41	94.52	38.46
CJ86	92.92	98.46	94.62	38.34
CJ91	91.19	98.32	94.25	38.55
CJ94	97.15	98.36	94.29	38.58

**Table 4:** Genomic distribution of SNP and InDel annotation effects.

Metric or Genomic Region	SNP		InDel	
	Count	Percent	Count	Percent
Unique variants	236,808,965	n/a	12,914,933	n/a
Annotation effects	265,985,527	100.000%	15,266,062	100.000%
Exonic	2,173,263	0.817%	78,919	0.517%
Intergenic	225,301,960	84.705%	11,951,105	78.285%
Intronic	8,895,472	3.344%	827,243	5.419%
Splicing	100,164	0.038%	8984	0.059%
Upstream	14,570,706	5.478%	1,153,823	7.558%
Downstream	14,534,928	5.465%	1,190,506	7.798%
UTR5	135,771	0.051%	17,023	0.112%
UTR3	273,263	0.103%	37,682	0.247%
Other	n/a	n/a	777	0.005%

Note: Unique-variant counts and annotation-effect counts use different units; a single variant may generate multiple effects because of transcript-level annotation. Percentages are based on annotation effects.

**Table 5:** SNP base-change counts and transition/transversion statistics.

Reference Base	A	C	G	T
A	0	5,765,445	23,117,384	7,942,689
C	9,855,193	0	5,269,816	66,380,241
G	66,403,416	5,271,890	0	9,936,039
T	7,992,934	23,095,976	5,777,942	0
Unique SNP sites	236,808,965			
Site-level transitions	178,997,017			
Site-level transversions	57,811,948			
Site-level Ts/Tv	3.0962			
SnpEff raw transitions	5,061,233,502			
SnpEff raw transversions	1,198,626,205			
SnpEff raw Ts/Tv	4.2225			

Note: The matrix summarizes 236,808,965 unique SNP sites. The SnpEff raw Ts/Tv ratio represents observed SNP events and therefore uses a different counting level.

**Table 6:** Predicted functional impacts of SNP and InDel annotation effects.

Type (Alphabetical Order)	SNP		InDel	
	Count	Percent	Count	Percent
HIGH	95,055	0.04%	58,486	0.38%
LOW	871,286	0.33%	7510	0.05%
MODERATE	1,326,552	0.50%	21,930	0.14%
MODIFIER	263,692,634	99.14%	15,178,136	99.42%

## Data Availability

The data presented in this study are available from the corresponding author upon reasonable request. The data are not publicly available because they form part of an ongoing germplasm evaluation and breeding program and have not yet been deposited in a public repository.

## Supplementary Materials

The following supporting information can be downloaded at: https://www.mdpi.com/article/10.3390/ijms27219828/s1.
