# Supplementary material for: Phenotypic Diversity and Genome-Wide Association Analysis of Decaploid Camellia hainanica Germplasm from Hainan Island
Source: Int J Mol Sci. 2026 Nov 2;27(21):9828. doi: 10.3390/ijms27219828 (PMC13763237; doi:10.3390/ijms27219828)
Supplement: Supplementary file 1 [file ijms-27-09828-s001.zip › ijms-4607357-supplementary.pdf]

Supplementary Materials

Phenotypic Diversity and Genome-Wide Association Analysis of Decaploid Camellia hainanica Germplasm from Hainan Island

Table S1. Phenotypic traits and measurement procedures.

Code	Trait	Type	Unit	Procedure
FLD	Fruit length	Quantitative	cm	Caliper; mean of 10 fruits
TDF	Fruit diameter	Quantitative	cm	Transverse diameter; 10 fruits
FSI	Fruit shape index	Quantitative	dimensionless	Fruit length / fruit diameter
WSF	Single fruit weight	Quantitative	g	Balance; 10 fruits
VN	Locule number	Quantitative	count	Counted in 10 fruits
PT	Pericarp thickness	Quantitative	cm	Caliper; 10 fruits
SN	Seed number	Quantitative	count per fruit	Counted in 10 fruits
FSW	Fresh seed weight	Quantitative	g per fruit	Balance; seeds from each of 10 fruits
NP	Petal number	Quantitative	count	Counted in 10 flowers
FD	Flower diameter	Quantitative	cm; mm in GWAS	Caliper; 10 flowers
PL	Petal length	Quantitative	cm	Caliper; 10 flowers
PW	Petal width	Quantitative	cm	Caliper; 10 flowers
PASD	Petal tip split depth	Quantitative	cm	Caliper; 10 flowers
NFPUA	Flower density	Quantitative	flowers per 0.25 m^2	Mean of 3 canopy quadrats, 0.5 × 0.5 m
HT	Tree height	Quantitative	m	Field height record; instrument not recorded
CW	Crown width	Quantitative	m	Field width record; instrument not recorded
DBH	Diameter at breast height	Quantitative	cm	Circumference in mm / (10 × π)
FOBH	First branch height	Quantitative	cm	Field height record; instrument not recorded
LL	Leaf length	Quantitative	cm	Caliper; 10 mature leaves
BW	Leaf width	Quantitative	cm	Caliper; 10 mature leaves
LA	Leaf area	Quantitative	cm^2	2/3 × leaf length × leaf width
PI	Leaf shape index	Quantitative	dimensionless	Leaf length / leaf width
FC	Fruit color	Qualitative	category	LY/T 2742-2016 categories in Table 2
FS	Fruit shape	Qualitative	category	LY/T 2742-2016 categories in Table 2
KS	Kernel shape	Qualitative	category	LY/T 2742-2016 categories in Table 2
SCC	Seed coat color	Qualitative	category	LY/T 2742-2016 categories in Table 2
FT	Flower type	Qualitative	category	Single / semi-double / double
DAC	Flower color	Qualitative	category	White with red spots / pure white
FOC	Leaf surface color	Qualitative	category	Yellow-green / green / dark green

Code	Trait	Type	Unit	Procedure
LE	Leaf margin	Qualitative	category	Four serration grades in Table 2
BS	Leaf shape	Qualitative	category	Five shape categories in Table 2

The 31 traits comprise 22 quantitative and 9 qualitative traits. Measurements were summarized at the accession level. NP, FD, VN, and NFPUA were used for GWAS; the other traits were used for phenotypic characterization. Units match main-text Tables 1 and 2, except that FD was entered in GWAS in mm. DBH was calculated from trunk circumference as circumference in mm/(10 × π). Instrument details not retained in the field records are identified rather than inferred.

Table S2. The 30 resequenced accessions, phenotypes, and observed phenotypic coverage.

ID	Formal GWAS	NP	FD (mm)	VN	NFPUA	Observed phenotype coverage
CJ1	Yes	5.10	72.20	3.00	23.00	VN lower decile; NFPUA upper decile
CJ6	Yes	5.00	65.64	3.00	9.33	NP lower decile; VN lower decile
CJ7	Yes	5.00	73.89	3.10	8.33	NP lower decile
CJ11	Yes	8.00	92.73	3.00	10.67	FD upper decile; VN lower decile
CJ17	Yes	6.50	77.10	3.20	9.67	Intermediate values for the four reported traits
CJ19	Yes	5.00	63.99	3.50	7.00	NP lower decile; FD lower decile
CJ20	No	8.40	75.10	3.60	4.67	NP upper decile; NFPUA lower decile
CJ21	Yes	7.50	82.12	3.80	16.00	Intermediate values for the four reported traits
CJ22	Yes	5.50	76.79	3.00	12.00	VN lower decile
CJ28	Yes	8.00	62.90	4.40	8.67	FD lower decile; VN upper decile
CJ39	Yes	8.20	94.20	4.70	11.00	NP upper decile; FD upper decile; VN upper decile
CJ57	Yes	7.80	69.09	3.60	20.33	Intermediate values for the four reported traits
CJ60	Yes	6.60	75.41	3.88	22.00	NFPUA upper decile
CJ61	Yes	7.70	60.80	4.70	2.00	FD lower decile; VN upper decile; NFPUA lower decile
CJ63	Yes	8.00	68.50	4.10	10.33	Intermediate values for the four reported traits
CJ66	Yes	8.10	60.86	4.30	8.67	FD lower decile; VN upper decile
CJ67	Yes	5.50	74.75	3.00	5.00	VN lower decile
CJ68	Yes	6.20	74.27	3.40	17.00	Intermediate values for the four reported traits
CJ72	Yes	7.20	56.54	4.50	18.75	FD lower decile; VN upper decile
CJ73	Yes	7.70	100.62	3.40	7.67	FD upper decile
CJ79	Yes	7.80	68.84	3.60	7.00	Intermediate values for the four reported traits
CJ80	Yes	8.00	102.12	3.70	31.33	FD upper decile; NFPUA upper decile
CJ81	Yes	8.30	87.74	3.30	9.00	NP upper decile
CJ86	Yes	8.00	94.50	3.40	13.00	FD upper decile
CJ91	Yes	6.00	75.80	4.20	10.00	Intermediate values for the four reported traits
CJ94	Yes	8.10	73.38	4.30	6.67	VN upper decile
CJ103	Yes	5.40	55.84	3.20	16.00	FD lower decile
CJ106	Yes	5.20	93.82	3.20	10.67	FD upper decile
CJ107	Yes	6.60	75.06	3.00	28.33	VN lower decile; NFPUA upper decile
CJ110	Yes	8.50	65.94	3.70	8.00	NP upper decile

CJ codes identify individual seed-grown trees; field, phenotype, and sequencing identifiers correspond directly. Values are accession-level phenotypes: FD in mm; NP and VN as mean counts; NFPUA as mean flowers per 0.25 m^2. Thirty accessions were resequenced. The table records their association-analysis status, and Figure 6 presents PCA of the resequenced panel.

The coverage column retrospectively describes measured percentiles in the 100-accession collection. “Lower decile” and “upper decile” denote the lower and upper 10% ranges, respectively; these are not recovered accession-specific historical selection notes or proof that thresholds were prespecified. No direct genetic-diversity comparison with the 70 unsequenced accessions is available.

Table S3. Gene annotations for the nine associated regions.

Trait and reference SNP	Gene model	Distance (bp)	Gene annotation
NP<br>Chr3:177,291,111	YC.01G0021180-2C	29,373	C2-containing protein
NP<br>Chr8:74,402,962	YC.02G0008720-2D	68,121	Ras-superfamily small GTPase (Ras/Roc/Arf-family matches)
NP<br>Chr21:182,770,806	YC.06G0025770-1A	9,697	Aquaporin-family protein
FD<br>Chr39:81,443,240	YC.10G0008000-2P	53,755	Agglutinin-like protein
NFPUA<br>Chr2:120,330,864	YC.01G0013500-1PB	23,402	TAXi-like protein; aspartyl-protease-family match
NFPUA<br>Chr58:76,638,829	YC.15G0008930-1B	28,556	SWEET-family sugar transporter
VN<br>Chr20:55,383,490	YC.05G0009190-3D	26,393	Cellulose-synthase-family protein
VN<br>Chr34:3,861,510	YC.09G0001060-2B	17,528	EF-hand calcium-binding protein
VN<br>Chr36:181,338,542	YC.09G0022670-3D	17,301	NOT2/NOT3/NOT5-family protein

Coordinates and gene models refer to the tetraploid Camellia oleifera assembly GWHERBT00000000. Gene annotations are reported for reference genes in the nine SNP-associated regions. Distance is measured from the SNP to the nearest boundary of the reported gene.

Protein annotations were assigned by sequence comparison against Pfam, with redundant matches summarized together. These annotations provide preliminary candidates for further study of the associated regions.

Reference assembly: https://ngdc.cncb.ac.cn/gwh/Assembly/83690/show. Pfam: https://www.ebi.ac.uk/interpro/entry/pfam/.

Table S4. Allele frequencies, additive effects, standard errors, and 95% confidence intervals of the nine reported trait-associated SNPs.

Trait	Reference SNP	REF / ALT	ALT AF	MAF	β ALT	SE	95% CI
NP	GWHERBT00000003: 177,291,111	T / C	0.4042	0.4042	+0.230294	0.025254	[0.178283, 0.282305]
NP	GWHERBT00000008: 74,402,962	A / G	0.3346	0.3346	-0.240167	0.021304	[-0.284043, -0.196291]
NP	GWHERBT00000021: 182,770,806	A / G	0.225	0.225	-0.274999	0.029919	[-0.336619, -0.213380]
FD	GWHERBT00000039: 81,443,240	T / C	0.3444	0.3444	+3.937732	0.423862	[3.064771, 4.810693]
NFPUA	GWHERBT00000002: 120,330,864	C / T	0.2222	0.2222	+2.103141	0.232115	[1.625091, 2.581190]
NFPUA	GWHERBT00000058: 76,638,829	G / C	0.1423	0.1423	+2.742969	0.297253	[2.130766, 3.355173]
VN	GWHERBT00000020: 55,383,490	C / T	0.3217	0.3217	+0.128233	0.014123	[0.099145, 0.157321]
VN	GWHERBT00000034: 3,861,510	G / A	0.069	0.069	+0.313949	0.034203	[0.243506, 0.384391]
VN	GWHERBT00000036: 181,338,542	G / A	0.1111	0.1111	+0.219769	0.023205	[0.171978, 0.267560]

REF, reference allele; ALT, alternative allele; AF, allele frequency; MAF, minor allele frequency; SE, standard error; CI, confidence interval. ALT AF = total observed ALT copies/(10 × non-missing genotypes); MAF = min(ALT AF, 1 − ALT AF). Frequencies were calculated before imputation. β ALT denotes the additive effect per ALT copy estimated using population-mode imputation, expressed in mm (FD), mean counts (NP and VN), or flowers per 0.25 m^2 (NFPUA). Model-based 95% CIs were calculated using t distributions without adjustment for SNP selection. Internal checks supported consistent effect directions.
